# *Helicobacter Pylori* Infection among Patients Attending the Gastroenterology Department in Tertiary Care Hospital, Kathmandu, Nepal

**DOI:** 10.1155/2022/1508473

**Published:** 2022-11-10

**Authors:** Tulasi Bhandari, Ujjwal Laghu, Prem Ratna, Rajani Shrestha

**Affiliations:** ^1^Department of Microbiology, St. Xavier's College, Tribhuvan University, Kathmandu, Nepal; ^2^Grande International Hospital, Dhapasi, Kathmandu, Nepal

## Abstract

*Helicobacter pylori* is one of the most pathogenic organisms that cause gastritis, peptic ulcer, gastric adenocarcinoma, and mucosa-associated lymphoid tissue (MALT) lymphoma in humans. The main aim of this study was to determine the *H. pylori* infection among patients undergoing upper GI endoscopy and to compare the efficacy of the diagnostic method of *H. pylori* infection including invasive tests (biopsy-based tests like the rapid urease test (RUT), direct smear, and culture) and the noninvasive test (HpSA). A total of 100 stool samples and 200 gastric biopsy specimens were collected (2 samples from each patient) from June to November 2019. Stool samples were processed for the detection of an *H. pylori* stool antigen (HpSA) by a kit method. One biopsy specimen was processed for the RUT, and another was transported to the laboratory in an Eppendorf tube containing normal saline for preparation of the smear and culture. Out of 100 participants, 26% were found to be *H. pylori* positive by the RUT, 11% by the direct smear, 6% by the culture, and 17% by the stool antigen test. The prevalence of *H. pylori* infection was found to be 14%, considering at least two of the three biopsy-based tests that gave positive results. *H. pylori* infection was found to be higher in the age group of 46–55 years. The overall prevalence of *H. pylori* infection was higher in gastric ulcer cases, followed by erosive pangastritis and gastroduodenitis. Tea drinking habits and the frequency of meal consumption more than twice a day were found to be significantly associated with *H. pylori* infection (*P* < 0.05). Hence, the RUT was found to be more efficient than the direct smear and the culture method for finding *H. pylori* in the biopsy sample. However, none of these methods can be considered to be the gold standard alone. Thus, the RUT combined with other tests is preferable for the detection of *H. pylori*.

## 1. Introduction

*Helicobacter pylori* (*H. pylori*) is a Gram negative, spiral, urease producing, and highly pathogenic microaerophilic flagellated bacterium which causes development of highly serious gastrointestinal disorders [1]. In 1982, Robin Warren and Barry Marshall discovered *H. pylori* as the causative agent of gastritis and peptic ulcers [2]. The organism produces the urease enzyme; the urease enzyme hydrolyses urea present in gastric juice to ammonia and carbon dioxide [3]. Infection with this organism is associated with gastritis and peptic ulcer diseases, which, when improperly managed, may eventually result in the development of gastric adenocarcinoma and mucosa-associated lymphoid tissue (MALT) lymphoma [4]. The second leading cause of cancer-related death in the world is gastric adenocarcinoma [5]. For that reason, H. *pylori* has been characterized as a group I human carcinogen since 1994 by the World Health Organization (WHO) [6, 7].

Most patients infected with *H. pylori* show no clinical symptoms, and the infection often persists without any clinically evident disease. The reason for this alteration may be bacterial pathogenicity and host susceptibility. *H. pylori* infection is associated with 90%–95% of patients with duodenal ulcers and 70% of those with gastric ulcers. Therefore, it is the main etiological agent of peptic ulcer diseases associated with or without upper gastrointestinal bleeding. Additionally, in all parts of the world, *H. pylori* is the strongest known risk factor for gastric cancer and mucosa-associated lymphoid tissue (MALT) lymphoma. The relative risk of gastric cancer in an *H. pylori*-positive group is 1.7–5.3 times higher than that in an *H. pylori*-negative group [8]. However, only 10%–20% of infected patients develop severe diseases during their lifetime [9]. In the global population, approximately 50% of infected *H. pylori* varied by prevalence, age, country, ethnic background, and socioeconomic condition. The worldwide population is affected by the gastric *H. pylori* infection. In developing countries, 70%–90% of the population is infected with *H. pylori* infection, which is mostly acquired during childhood, whereas it is lower in developed countries, ranging between 30% and 40% [10]. The exact route of transmission is still unknown, and person-to-person transmission is most likely via either oral-oral or fecal-oral routes [11].

*H. pylori* infection can be diagnosed by invasive and noninvasive methods, each having its advantages and disadvantages. Invasive methods require an endoscopic biopsy of the gastric mucosa and can be tested by various methods, like the rapid urease test (RUT), histology, culture, and polymerase chain reaction (PCR), whereas noninvasive tests include the stool antigen test, serology, and urea breath test (UBT). The choice of a specific test is determined by several factors, like clinical conditions of the patient, cost of the test, and its sensitivity and specificity [12].

*H. pylori* infection is acquired early in life (almost always before the age of 10 years), and once acquired, it generally persists for life unless treated by antibiotic therapy [13]. Eradication of the *H. pylori* infection cures the ulcer, usually permanently, since reacquisition of this bacterium infection is rare [14]. The current established treatments for *H. pylori* infection are numerous and include triple and quadruple therapy, both of which utilize two antibiotics (metronidazole, amoxicillin, tetracycline, or clarithromycin) in addition to either a proton pump inhibitor (PPI) (triple therapy) or a PPI and bismuth (quadruple therapy). The lower incidence of infection with *H. pylori* has been associated with the consumption of many foods of vegetal origin, including wine and green tea which are rich in phytochemicals such as flavones, isoflavones, flavonols and flavanols, anthocyanidins, tannins, and stilbene derivatives. Taken together, it is necessary to find new therapies that would help eradicate *H. pylori* infection and prevent gastric cancer [15].

Gastritis is one of the major health problems in developing countries like Nepal in comparison to developed countries. It might be due to lower socioeconomic conditions, poor sanitation, and poor hygiene practice. Therefore, early detection of *H. pylori* infection is important to eradicate *H. pylori* infection. This study could help clinicians choose appropriate diagnostic methods for early diagnosis of *H. pylori* infection and prevent the development of peptic and duodenal ulcers in patients. This study will also help know the possible risk factors of *H. pylori* infection.

## 2. Methods

The research was a hospital-based, cross-sectional, prospective study that was conducted in the Microbiology Laboratory of Grande International Hospital, Dhapasi, Kathmandu, from June to November 2019. A total of 100 stool samples and 200 gastric biopsy specimens (2 specimens from each patient) were collected from 100 patients and processed during the study. All relevant clinical and sociodemographic data were collected using a structured and pretested questionnaire before collecting the samples.

### 2.1. Inclusion Criteria

Patients having upper GI tract infections were included.

### 2.2. Exclusion Criteria

Patients who were treated with any antibiotics, colloidal bismuth compounds, and proton pump inhibitors (PPIs) within the last two weeks and had a history of gastrectomy were excluded.

A history of risk factors like sociodemographic risk factors and habitual risk factors was taken to see whether any statistical significance with *H. pylori* infection was there or not.

Written informed consent was obtained from all the patients before endoscopy and sample collection. Approval was obtained from the Ethical Review Board of the Nepal Health Research Council (Reg. no. 586/2019) before the initiation of this study.

#### 2.2.1. Endoscopy and Biopsy Sampling

Endoscopy was performed by expert gastroenterologists using an Olympus video endoscope. Two gastric biopsies were taken from each patient from the gastric antrum and corpus. In which, the first was directly inoculated into the well of a rapid urease test (RUT) kit. The second was placed into a sterile Eppendorf tube containing 0.5 ml of normal saline to maintain humidity. The samples were transported to the microbiology laboratory as soon as possible. The samples were processed within 4 hours.

### 2.3. Rapid Urease Test (RUT) of Biopsy Specimens

Immediately after collection, one gastric biopsy specimen was directly put into the well of the kit and was processed as per the instructions mentioned in kit GASTRO CURE SYSTEMS, Kolkata, India. It was incubated at room temperature and examined within 1 hour for color change. Immediately, Urease positive isolates changed the color of the medium from yellow to pink.

### 2.4. Smear Preparation and Culture

The second biopsy specimen was used for the culture. The sample was crushed into small pieces using a sterile scalpel blade. One of the small crushed samples was used for gram staining. Then, other crushed biopsy samples were inoculated into chocolate agar and Columbia blood agar supplemented with 7% sheep blood. A microaerophilic environment was created in the candle jar system. To ensure high humidity, tissue papers soaked in sterile water were placed in an open Petri dish. Inoculated plates, the open Petri dish containing wet tissue papers, and a candle were placed in a glass jar. The jar was immediately covered with its lid, completely sealed with Vaseline, and incubated at 37°C for up to 7 days. After incubation for 2 days, the plates were examined for growth daily. If no growth of *H. pylori* was observed after incubation for 7 days, the plates were discarded.

The growth of *H. pylori* isolates was confirmed by standard microbiological techniques like colony morphology, gram staining, and biochemical tests (catalase, oxidase, and urease tests).

### 2.5. Stool Antigen Assay (HpSA)

Stool samples were analyzed using the Fastep *H. pylori* Antigen Rapid Test device (feces) (Polymed Therapeutics, Inc, 3040 Post Oak Blvd., Ste 1110 Houston, TX 77056, USA) by following the manufacturer's instructions. Stool specimens were collected by inserting an applicator stick into at least 3 different sites of feces to collect approximately 50 mg of feces and transferred into a specimen collection tube containing extraction buffer. 3 drops of the solution were transferred into the specimen well (*S*) of the test device. The result was read in 10 minutes after dispensing the specimen. The appearance of two colored bands as a control band (*C*) and test band (*T*) on the membrane indicated the positive result, and one line in the control region indicated the negative result. Disappearance of the control line indicated the invalid result.

### 2.6. Data Analysis

Data were entered and analyzed using the SPSS version 16 software package. A *p* value <0.05 was considered statistically significant.

## 3. Results

Out of 100 patients, the highest prevalence of *H. pylori* positivity was found in 26% by the rapid urease test, followed by 11%by the direct smear and 6% by the culture, respectively. Additionally, *H. pylori* infection was detected in 17% of patients by performing the noninvasive test like the stool antigen test. The overall prevalence of *H. pylori* infection was found to be 14%. A patient was considered to be positive for *H. pylori* infection when at least two of these three biopsy-based tests (rapid urease test, direct smear, and culture) gave a positive result.

Among the 14 total positive cases, 2 cases were positive by the RUT, direct smear, and culture. Similarly, 8 cases were positive by two tests: RUT and direct smear. Three cases were positive by two tests: RUT and culture, and 1 case was positive by the direct smear and the culture. Statistically, there was a significant association between positivity for *H. pylori* and different combinations of biopsy-based tests (*p* = 0.035). The result is shown in Table 1.

In total positive cases, the prevalence of *H. pylori* infection was found to be 15.2% in males and 12.9% in females. It was not found to be statistically significant (*p* value = 0.746). The age of patients ranged from 16 to 83 years. Among positive cases, the highest percentage (20%) of positive cases of *H. pylori* infection was found in the age group of 46–55 years.

Among the 26 rapid urease positive cases, 11 (42.30%) cases were stool antigen positive. Out of 74 rapid urease negative cases, only 6 (8.10%) cases were stool antigen positive. Statistically, there was a significant association between the rapid urease test (RUT) and the *H. pylori* stool antigen (HpSAg) detection test (Table 2).

Distribution of *H. pylori* according to different diagnoses is presented in Table 3. The overall prevalence of *H. pylori* infection was higher in gastric ulcer cases (*n* = 2, 100%), followed by erosive pangastritis (*n* = 2, 50%) and gastroduodenitis (*n* = 2, 50%). Among various gastric disorders, gastric ulcers were only found to be significantly associated with *H. pylori* infection (*p* ≤ 0.001).

Among various habitual risk factors, tea drinking habits and the frequency of meal consumption more than twice a day were found to be significantly associated with *H. pylori* infection (*p* = 0.048) and (*p* = 0.030), respectively, as shown in Table 4.

## 4. Discussion

*Helicobacter pylori* infection was detected by performing biopsy-based methods, including the rapid urease test, direct smear, and culture by 26%, 11%, and 6%, respectively. Additionally, *H. pylori* infection was detected in 17% of patients by performing a noninvasive test like the stool antigen test. In this study, the prevalence of *H. pylori* infection was found to be 14% with the consideration that at least two of the three biopsy-based tests (RUT, direct smear, and culture) gave a positive result. Numerous studies are carried out in Nepal related to *H. pylori infection*. The prevalence of *H. pylori* infection as 16% was reported by Ansari et al. by stool antigen test [16]. Another report by Pilli and Kirani showed that 14% of patients were culture positive, 18% were Gram stain positive, 36% were rapid urease test positive, and 42% were serology IgG antibody positive for *H. pylori* [17]. The direct Gram-stained smear is an invasive, highly specific, rapid screening test, and in a resource-limited laboratory, a need for additional testing of positive specimens may be avoided.

Culture-positive biopsies were relatively low at 6% (6/100) as compared to the rapid urease test, direct smear, and stool antigen test results. This difference in positive results of the culture may be due to the overgrowth of contaminants. A single culture plate revealed a mixed bacterial population containing two or more different types of organisms like Staphylococcus aureus, *Pseudomonas* species, *Candida* species, *Klebsiella* species, and *Proteus* species. Hence, due to its fastidious, slow growing nature, the growth of *H. pylori* is difficult to detect. The sensitivity is lower for several reasons, like low colonization because of the patchy distribution of bacteria in the stomach, contamination of biopsy forceps, decreased bacterial viability, atmospheric conditions, and varied culturing methods. In this study, selective media have not been used. Selective media such as Skirrow's supplement in the culture media is important to inhibit the growth of other bacterial populations and contaminants and for better yield of the growth of *H. pylori*. This study is consistent with the study laid by Sharma et al. who isolated nine specimens of *H. pylori* out of 100 by culturing on nonselective medium supplemented with 7% sheep blood [18]. Culturing is costlier than other options, is technically challenging, and requires strict conditions [19]. Furthermore, in low resource settings, the RUT and the stool antigen may play a vital role in clinical management of the infection.

In this study, the highest prevalence was found in patients in the age group of 46–55 years. There was no statistically significant association between *H. pylori* and age category of patients. Age distribution of *H. pylori* infection did not show any trend toward an increase or decrease in infection with the advancing age, which is in accordance with Shakya et al. and Agarwal et al. who also reported similar findings [20, 21].

In the present study, the prevalence of *H. pylori* infection was slightly more among males (15.2%) than females (12.9%) but did not show a significant association (*p* = 0.746) between gender and presence of *H. pylori* infection. As reported in other studies males were at higher risk than females for the colonization of *H. pylori* but the association between *H. pylori* and gender was not found significant [20, 22]. The reason for the observed difference was not known, but better hygienic practice may be the reason for lower prevalence in females [20].

In this study, among the 26 RUT positive cases, 11 (42.30%) cases were stool antigen positive. Out of 74 RUT negative cases, only six (8.10%) cases were stool antigen positive. Statistically, there was a significant association between the rapid urease test (RUT) and the *H. pylori* Stool antigen (HpSAg) detection test. The RUT is an invasive method and considered a gold standard. This test is cheap and allows for rapid detection. The test may result in false negative if the patient has recently used antimicrobial agents, like PPIs or bismuth-containing compounds [23], and also may be due to the complete absence of *H. pylori* or the patchiness of the organism [24]. Many studies have claimed that the stool antigen test is useful for the primary diagnosis and post-treatmentfollow-up of *H. pylori* infection [25]. However, due to its low validity in predicting *H. pylori* infection, the stool antigen test could be an important diagnostic test in follow-up patients, like the urea breath test, which in turn decreases the number of patients requiring invasive tests and cost as well [26].

Out of total cases, antral gastritis and erosive gastritis were the most common endoscopic findings, whereas the overall prevalence of *H. pylori* infection was higher in gastric ulcer cases and found to be significantly associated with *H. pylori* infection (*p* ≤ 0.001).

Among various ethnic groups, Madhesi was found to be significantly associated with *H. pylori* infection (*p* = 0.009). In contrast, Miftahussurur et al. did not show a significant association between the ethnic group and presence of *H. pylori* infection [27]. The reasons for higher prevalence of infection among Madhesi are unknown. The observation suggests there may be genetic factors responsible for enhanced susceptibility to *H. pylori* infection and ethnic lifestyle differences or sanitation practices [28].

Among various risk factors, tea drinking habit was found to be significantly limt the colonization of *H. pylori* (*P* = 0.048). This study is similar to the study done by Ansari et al. [16]. Tea catechins have antibacterial activity against various food-borne pathogenic bacteria. Thus, for eradication of *H. pylori*, it seems reasonable to explore the possibility of using tea catechins and harmless compounds extracted from green tea [29]. Pyrogallol and gallate substituent groups of catechin compounds are an essential element of antimicrobial activity [30]. Natural remedies components such as green tea could be further used for prevention and treatment of *Helicobacter*-induced gastritis in humans [31].

In current study, the frequency of meal consumption for more than two times per day significantly limited the *H. pylori* colonization (*p* = 0.030). This was comparable to the results of the study by Ansari et al. [16]. Alcohol drinking, cigarette smoking, and type of food consumption (vegetarian and nonvegetarian) had no significant association with *H. pylori* infection (*p* > 0.05). This was consistent with the findings by Dilnessa and Amentie [32]. In this study, the absence of an association might be due to less number of alcohol users, and the type and the amount of alcohol consumed affect the association.

## 5. Conclusion

The prevalence of *H. pylori* infection was 14%. A reliable test to detect *H. pylori* infection is essential, but none of the tests available is suitable for all situations. The bacteriological culture is not suitable for routine diagnosis because of the fastidious nature of the organism. The RUT is best considered a screening test, but not a gold standard test for *H. pylori*. The HpSA test is a rapid, easy to perform, noninvasive test that decreases the number of patients requiring invasive tests and cost as well. However, due to its low validity in predicting *H. pylori* infection, the stool antigen test could be an important diagnostic test in follow-up patients and might be a reliable test in settings where endoscopy service is not available. Gastric ulcers were found to be significantly associated with *H. pylori* infection. Among various risk factors, tea drinking habits and consumption of meal more than twice a day were significantly associated with *H. pylori* infection.

## Figures and Tables

**Table 1:** Positivity for *H. pylori* with different combinations of biopsy-based sample tests.

Test	*H. pylori* positive	Chi-square	*p* value
RUT + smear + culture positive	2		
RUT + smear positive	8	8.586	0.035
RUT + culture positive	3		
Smear + culture positive	1		
Total	**14**		

^*∗*^Gold standard: any two tests taken as positive.

**Table 2:** Relationship of the rapid urease test (RUT) with *H. pylori* stool antigen (HpSAg) detection test in various gastric illness patients *n* = 100.

Test	Number of cases	HpSAg positive	HpSAg negative	Chi-square	*p* value
RUT positive	26	11 (42.30)	15 (57.69)		
RUT negative	74	6 (8.10)	68 (91.89)	2.000	≤0.001
Total	**100**	**17**	**83**		

**Table 3:** Distribution of *H. pylori* according to different diagnoses.

Endoscopy findings	Number tested *n* = 100	*H. pylori*positive *n* = 14 (%)	Chi-square	*p* value
Antral gastritis	48	7 (14.6)	0.026	0.872
Erosive gastritis	37	3 (8.1)	1.693	0.193
Erosive pangastritis	2	1 (50)	2.197	0.138
Gastroduodenitis	2	1 (50)	2.197	0.138
Gastric ulcer	2	2 (100)	12.536	≤0.001
Duodenitis	1	0 (0)	0.164	0.685
Duodenal ulcer	2	0 (0)	0.332	0.564
Normal	5	0 (0)	0.857	0.355
Esophageal ulcer	1	0 (0)	0.164	0.685

**Table 4:** Habitual risk factors.

Participant characteristics	Total cases *n* = 100	Positive cases *n* = 14 (%)	Chi-square	*p* value
Tea drinking habit			3.908	0.048
Yes	72	7 (9.7)		
No	28	7 (25)		
Smoking habit			0.565	0.452
Yes	22	2 (9.1)		
No	78	12 (15.4)		
Alcohol drinking habit			0.056	0.813
Yes	26	4 (15.4)		
No	74	10 (13.5)		
Type of food consumption			0.036	0.850
Vegetarian	16	2 (12.5)		
Nonvegetarian	84	12 (14.3)		
Frequency of meal consumption per day			4.729	0.030
Two times	32	8 (25)		
More than two times	68	6 (8.82)		

## Data Availability

The data used to support the findings of this study are added in the article.
